# Round the Hospitals

**Published:** 1916-11-25

**Authors:** 


### ROUND THE HOSPITALS.

On Thursday of last week the Military Hospital, Hampstead, was honoured by a visit from Their Majesties King George and Queen Mary, attended by the Countess of Minto and Commander Sir Charles Cust, R.N. Every ward was visited; the King and Queen chatted with most of the patients and asked many questions of the Commanding Officer and the matron, who conducted Their Majesties round. The visit lasted over an hour, and both patients and staff are most grateful for the kindly and practical interest thus shown in the work of the hospital.

---

Miss Rundle, the Secretary of the College of Nursing, desires to express her appreciation of the kindness and courtesy of matrons who are taking an infinity of trouble to hunt up the records of their graduates who may apply to register with the College, and to supply full particulars. Their prompt and ready courtesy, with the complete information they send, are a great service to the individual nurse and the profession as a whole. In addition to the name of the training school, the College requires three references from every applicant. It sometimes happens that an employer has completely forgotten the identity or qualifications of the nurse who may have given his or her name as a reference; and as an act of courtesy which at the same time guards against this risk, with the lengthy delays that may ensue, nurses should always write beforehand to ask permission to give a name as a reference. Hospital matrons and sisters can do a useful service by impressing this fact on their nurses and any would-be candidates for early registration with the College.

---

THE nursing profession generally, and members of the Royal British Nurses' Association particularly, will be interested to learn that the prize of £50 and the medal of the Institute offered by the Royal Sanitary Institute for the best thesis setting out a complete and practical scheme for maternity and child-welfare work, suitable for adoption by local authorities, has been won by Miss Isabel Macdonald, the secretary of the R.B.N.A., and Miss Kate Cropper Atherton, who submitted a joint essay. One hundred and thirty-seven essays were submitted, and were adjudicated upon by Prof. H. R. Kenwood, Dr. John Robertson, and Dr. S. W. Wheaton. Miss Atherton, like Miss Macdonald, is a trained nurse, and both hold a health visitor's certificate. We congratulate these ladies on their success, and are sure these congratulations will be echoed by members of the Royal British Nurses' Association, on whose behalf Miss Macdonald has worked so energetically and fruitfully since her appointment as secretary seven years ago.

---

AN elderly and much-travelled V.A.D. was discussing her experiences as a pupil nurse at one of the great London hospitals, and having made a few shrewd criticisms, went on to say that the thing which struck her most in hospital life was the etiquette. "It is stricter," she said, "than any court in Europe." Students of women's colleges and other institutions often betray astonishment at the etiquette demanded in hospitals, and it has been asked whether it is not a little overdone? The matron is kept so aloof from her sisters and nurses that may she not sometimes lose touch with them? The head of more than one women's college rows and plays cricket with the students, and yet is able to retain her authority. Would a matron necessarily lose hers if she unbent sometimes and met her nurses on common ground? If the matron of the future has lived a college life for a year and seen that the principal can meet her pupils on the common ground of mutual outside interests and yet not lose her position or allow loss of discipline, she may come to realise that in the social life of the nurse-training school it should be possible to develop a more human attitude, and to cast aside some of the artificial barriers which hinder a good understanding between teacher and pupil.

---

ANOTHER side of the question is that of the regulation of social relations between the medical and nursing staffs. There are still, unfortunately, some matrons whose only idea of regulating these relations is to forbid them altogether, with the result that clandestine flirtations inevitably take the place of simple friendly intercourse. It is otherwise in one large hospital, where the flat roof of a new wing has been set apart for recreation for the staff. Here supper-parties have been permitted on summer evenings on two conditions; that at least one of the sisters is always present, and that no one stays out after 10 P.M. The results have entirely justified the matron's confidence in her nurses.

---

THE paragraph as to a weekly day off duty in our issue of November 11 has brought us a communication from a matron of experience, insisting on what she regards as certain essentials which the matron should bear in mind when planning the times off. They are that the off-duty hours should be in daylight; that two seniors in the same ward should not be off duty together; that there should be a time-table of the off-duty hours, which should not be altered except under very special circumstances, and then only with the matron's sanction; that rigid punctuality should be insisted on; that classes or lectures should not be held during the nurses' off-duty hours. Strict attention to these points does much to enable the nurses to get the utmost value out of their off-duty time with the minimum of inconvenience to the hospital.

---

LIEUTENANT-COLONEL SIR J. KINGSTON FOWLER, K.C.V.O., M.D., F.R.C.P., has been appointed a member of the Supply of Nurses Committee, in place of Sir Frederick Treves, who has resigned.

For the College of Nursing and State Registration, see p. 169.

